# Computational Study of a Versatile Lipase for the Degradation of Polylactic Acid

**DOI:** 10.1021/acsomega.5c07809

**Published:** 2025-11-20

**Authors:** Carlos Murguiondo, Mario García de Lacoba, Valentina Acosta-Borreros, Jorge Barriuso, Alicia Prieto

**Affiliations:** Centro de Investigaciones Biológicas Margarita Salas (CIB-CSIC), Ramiro de Maeztu 9, Madrid, Community of Madrid ES 28040, Spain

## Abstract

Biocatalysis is an emerging and sustainable approach to depolymerize highly hydrophobic plastic polyesters such as poly­(lactic acid) (PLA), a bioplastic widely used in packaging and disposable items. Some enzymes, including lipases, cutinases, and proteases, have been described to hydrolyze PLA, but the activity strongly depends on stereochemistry and crystallinity. In this study, we explored the activity of the versatile lipase from*Ophiostoma piceae* (OPE) and three engineered variants (N81A, N94A, and N81/94A) on polylactic acid (PLA), comparing the experimental data with predictions from two computational methodologies, Thermal Titration Molecular Dynamics (a classical method) and the machine learning-guided XLPFE scoring function. Experimentally, mutant N81A showed the highest PLA hydrolytic activity, followed by WT OPE, with N94A and N81/94A being substantially less effective. This combined approach served to validate the reliability of these computational strategies for predicting enzyme interactions and highlights the importance of using long enough model substrates to guide future enzyme optimization.

## Graphical abstract

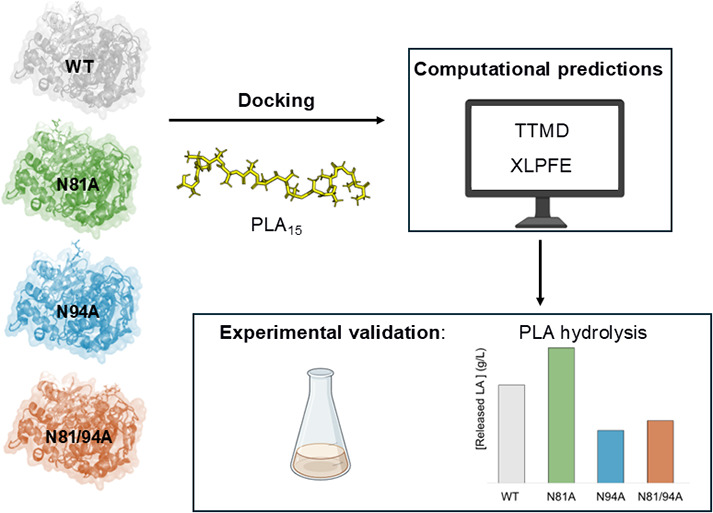

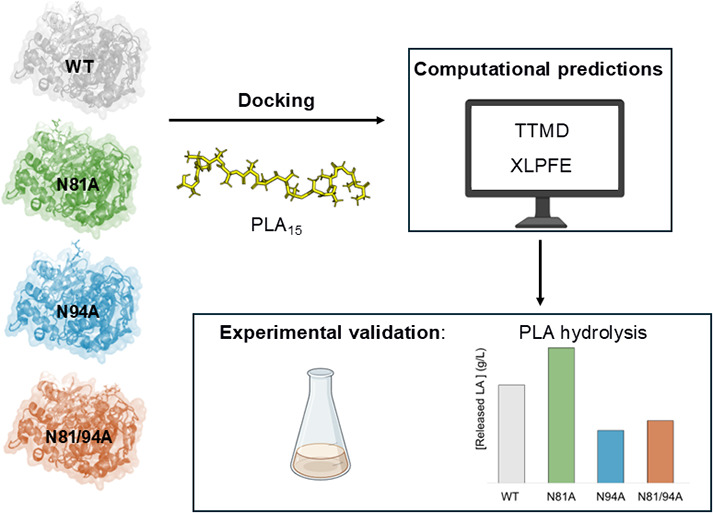

## 1 Introduction

The rapidly expanding field of biotechnology relies on the use of enzymes for a wide range of industrial applications. The biocatalytic degradation of synthetic polymers, like polylactide (PLA), is in the spotlight due to its widespread use and limited biodegradability. PLA is an aliphatic polyester composed of repeated units of lactic acid (LA) derived from renewable resources. Due to its biocompatibility and thermoplasticity, it is being used to replace poly­(ethylene terephthalate) (PET), polystyrene, or polyethylene in several applications in agriculture, transport, textile, medical, and packaging sectors.^1–5^ The current management of PLA waste primarily focuses on recycling and composting, and enzymatic hydrolysis emerges as a green solution^5,6^ for the recovery and reuse of oligomers and monomers.

PLA exists as three enantiomers: PLLA (containing only the l isomer), PDLA (containing only the d isomer), and PDLLA (containing both d and l isomers). The physical features of each of these polymers make them appropriate for different end uses.^5^ Several types of hydrolases have shown potential for PLA hydrolysis, with proteases being selective toward PLLA while lipase- and cutinase-type enzymes show stereopreference toward PDLA with varying efficiencies, and are also active on PDLLA.^7–9^ The enantiopure forms of PLA are semicrystalline, with high tensile strength and low elongation, while PDLLA is more amorphous, with its degree of crystallinity depending on the D/L ratio.^5,10^ These structural differences determine their suitability for distinct applications. PLLA, with its greater versatility and resistance, is commonly employed in industrial applications that demand high mechanical and thermal stability. In contrast, amorphous PDLLA is preferred in applications where enhanced biodegradability is required, such as disposable cutlery, cups, plates, and outdoor items.^11,12^ Moreover, as noted by Lim et al.,^13^ most commercial PLA products are manufactured as copolymers of PLLA and PDLLA. Although the geometry of lipases, with a lid region covering a hidden tunnel-like catalytic pocket, limits their efficiency at degrading PLA, they remain a subject of study due to their versatility, thermostability, and potential for improvement through enzymatic engineering.

The versatile lipase from the wood-saprophytic ascomycete fungus*Ophiostoma piceae* (OPE) has been described as an enzyme with wide substrate specificity and very efficient in hydrolysis and synthesis reactions.^14–16^ Structurally, OPE is an α/β-hydrolase with the catalytic triad Ser220-Glu352-His465 and a 37 amino acid lid region formed by an α-helix and two 3_10_ helices rich in hydrophobic amino acids.^17^ In its inactive state, the lid covers access to the active center, but in the presence of substrates, it opens leaving free access to the active site. Payá-Tormo et al.^18^ designed 3 mutated variants of the lid region of OPE with increased hydrophobicity (N81A, N94A, and the double mutant N81/94A) and characterized their catalytic properties against triglycerides and *p*-nitrophenol esters of different chain lengths. To understand the mechanistic basis behind the results, they performed Molecular Dynamics (MD) simulations and found that the substitution of asparagine for alanine in the variant N94A led to a larger hydrophobic area in the active form of the enzyme that allows better accommodation of the *p*-nitrophenyl butyrate (*p*NPB) substrate. They suggested that together with the increased hydrophobicity of the lid region, this would allow better stabilization of hydrophobic parts of ligands. Therefore, it is reasonable to think that these OPE mutants with more hydrophobic lids might exhibit enhanced activity toward hydrophobic and bulky polymers like PLA.

*In silico* approaches have shown to be very useful to significantly reduce the time and resources needed to characterize the catalytic properties of enzymes, but their experimental validation remains essential.^19^ Over the past decade, kinetic rates of protein–ligand binding have shown to correlate better with catalytic efficacy than thermodynamic parameters such as the equilibrium dissociation constant.^20^ However, on a molecular level, the protein–ligand binding/unbinding processes occur at much longer time scales than those of typical MD simulations, imposing severe restrictions in terms of affordable computational time and practical resources’ availability.^21,22^ Tools like DiffDock,^23^ a state-of-the-art MD docking tool, allow the accurate prediction of protein–ligand binding poses, while several computational methods, primarily rooted in classical approaches or machine learning (ML)-based models, are emerging to predict parameters such as stability, affinity, or binding poses of the protein–ligand complexes.^24,25^ Among the classical approaches, Thermal Titration Molecular Dynamics (TTMD)^26^ is a novel computational method that combines a series of MD simulations performed at progressively increasing temperatures with an analysis based on protein–ligand interaction fingerprints for the qualitative assessment of protein–substrate binding stability. On the other hand, ML models such as XLPFE have been developed to predict binding affinity and ligand poses with high accuracy. XLPFE^27^ integrates structural, energetic, and statistical descriptors within an ensemble learning framework for protein–ligand scoring and ranking.

One of the targets of the current work was to determine if these tools were also applicable to polymer hydrolysis, considering that DiffDock, TTMD, and XLPFE have been developed primarily for small-molecule ligands, which can limit their usefulness. This study evaluates for the first time their reliability to study the interaction of an enzyme and a polymeric substrate, benchmarking the computational predictions for PLA depolymerization with *in vitro* assays. PDLLA was used for the experiments since it is amorphous and, thus, more susceptible to enzymatic attack. Altogether, the methods evaluated offer insights into enzyme–substrate interactions and potential efficiencies.

## 2 Materials and Methods

### 2.1 Materials

Unless otherwise stated, all materials were bought from Sigma-Aldrich, including the substrate PDLLA with an *M*_w_ between 75 and 120 kDa (ref 67122).

### 2.2 Structure Modeling of Proteins and Ligands

The crystal structure of the open conformation of the OPE monomer (PDB 4BE9, chain A)^14^ was used as a *bona fide* starting structure for the modeling strategy. The 3D structures for the three mutants (N81A, N94A, N81/94A) were modeled applying a consensus prediction of the effects of mutations derived by the Site Directed Mutator (a statistical potential energy function) as a predictor of the effect of SNPs on the stability of proteins, and by mCSM (an ML method) to predict the effects of missense mutations based on structural signatures, both as implemented in DUET.^28^ Regarding the substrate, only PDLA models were used for computational simulations, given the preference of lipase-type enzymes toward this isomer and the impossibility to predict the distribution of d- and l-LA in PDLLA. This choice reduces the system’s structural and overall complexity, which is important to obtain clear results.^28^ The CHARMM-GUI polymer builder generator^29^ was employed to build PLA oligomer chains of 3 and 15 repeating d-LA units (PLA_3_ and PLA_15_, respectively). PLA_15_ was considered long enough to encompass interactions with the entire protein surface.

### 2.3 Docking

In order to assemble the protein–ligand structures for the different OPE (WT, N81A, N94A, N81/94A)-PLA (3-LA, 15-LA) complexes, an ML-based molecular semiflexible docking algorithm was applied as implemented by DiffDock v.1.1.3 (https://github.com/gcorso/DiffDock). This algorithm implements a pocket-level flexible docking approach, allowing ligand flexibility and side-chain flexibility within the binding pocket while maintaining the protein backbone mostly fixed.^23^

### 2.4 *In Silico* Predictions of the Properties of PLA Docking Complexes

The TTMD computer simulations under a GPU-processing environment were performed in the Spanish supercomputing cluster facility Finis Terrae III (CESGA, Node of the Spanish Supercomputing Network). Each TTMD simulation is associated with a mean squared (MS) coefficient (slope of the titration profile, plotted as the fingerprint-based scoring function vs temperature throughout the MD trajectory), which ranges from 0 (indicative of strong binding related to a high residence time of the starting binding pose) to 1 (indicative of weak binding through low residence time). For each ligand, five independent TTMD simulations were performed, and the average MS coefficient was then calculated based on three of them, discarding the highest and lowest values.^30^ Each MD simulation, both in the equilibration and the 160 ns production stage, was performed using an integration time step of 2 fs, keeping the temperature at a constant value of 310 K through a Langevin thermostat.

The XLPFE algorithm was implemented as a Conda environment on an in-house Linux computer server (32 physical cores, 500 GB RAM) and used to predict the ML-based scores for all of the distinct OPE-PLA complexes. In XLPFE, a higher score value is indicative of a higher binding affinity.

### 2.5 Protein Production and Purification

Proteins were produced using a methanol-inducible system in*Komagataella phaffii* (formerly *Pichia pastoris*) with a pPIC9 vector, concentrated and purified using an AKTA FPLC system and a HiTrap Octyl Sepharose FF Cartridge (GE Healthcare), as described previously.^18^ Purity and molecular masses of the denatured enzymes were confirmed by SDS-PAGE using 12% polyacrylamide gels. Protein concentration was measured with the Bradford assay.^31^ Esterase activity was measured using *p*NPB as described before.^32^

### 2.6 *In Vitro* Hydrolysis of PLA

PLA pellets with an *M*_w_ between 75 and 120 kDa (Sigma-Aldrich, ref 67122) were ground in a grinder (Analysenmühle A10, Janke & Kunkel, IKA Labortechnik) with a water-cooling system to avoid melting and recrystallization^33^ and passed through a 420 μm sieve to select small particles. Hydrolysis was conducted with 10 g/L PLA and 0.1 g/L of the corresponding purified enzyme in 100 mM Tris-HCl (pH 7) at 50 °C and 1000 rpm. These conditions were chosen based on preliminary experiments (data not shown). At the final time, the reactions were stopped by boiling the samples at 100 °C for 5 min to inactivate the enzymes. The released LA was determined by HPLC in an Agilent 1260 Infinity instrument equipped with an Aminex 87H column (Bio-Rad) and a refractive index detector (RID). The samples were analyzed at 55 °C, with 5 mM H_2_SO_4_ as the mobile phase at 0.5 mL/min. The peak from the LA was identified by its retention time and quantified using a calibration curve. All reactions were performed in triplicate.

## 3 Results and Discussion

### 3.1 Comparative Docking of Model Substrates Highlights the Impact of Substrate Length on Predictive Accuracy

The docking simulations offered structural insights into how each OPE variant interacts with PLA chains of 3 and 15 lactic acid units. While the predictions suggested that the PLA_15_ substrate exhibited distinct positions and orientations in each lipase variant (Figure 1), PLA_3_ displayed similar spatial arrangements in all variants (data not shown). This is likely due to the short length of the trimer, which is insufficient to replicate the interactions between PLA and the lid of the different OPE variants. In contrast, the 15-mer chain appears to be long enough to interact with the protein surface. This finding emphasizes the necessity of long model substrates for accurately studying the docking of polymers.

**Figure 1:**
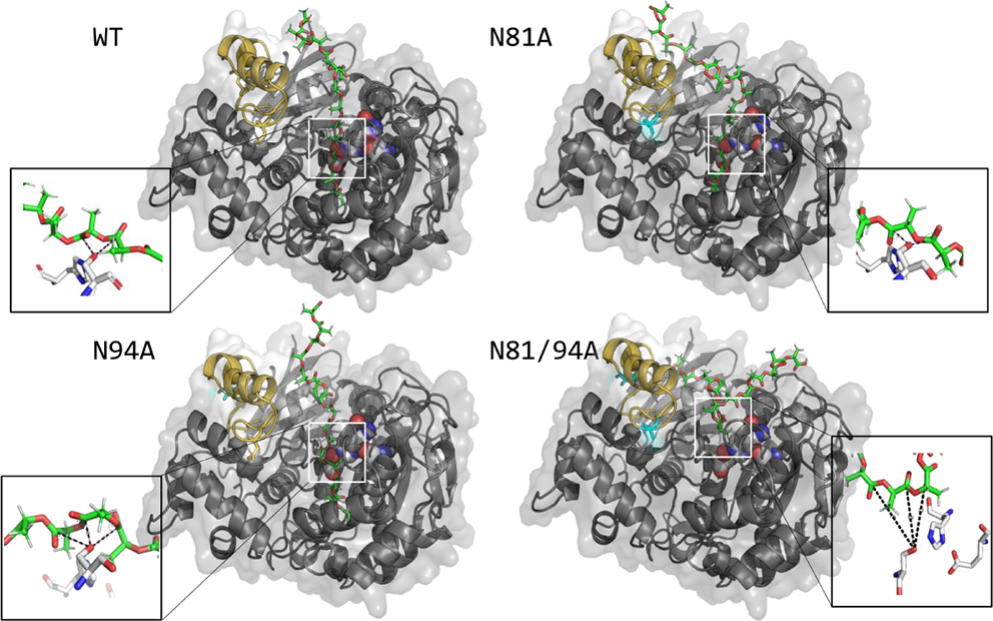
Docking of the open conformation of the OPE variants with PLA_15_. The lid is shown in yellow, with the mutations (N81A and/or N94A) in cyan. The catalytic triad (S220, E352, H465) is shown as spheres, and the ligand (PLA_15_) is shown as sticks (C in green). The zoomed-in windows show the interactions between the −OH group of the catalytic S220 and the carbonyl groups of PLA_15_.

As reported by Payá-Tormo et al.,^18^ the mutations studied in this work cause not only enhanced hydrophobicity but also significant conformational changes in the lid region of the three engineered variants, like the increased lid flexibility in mutant N81A (the double mutant has an intermediate lid flexibility between N81A and N94A) and the presence of a larger cavity in the lid area in the N94A variant. These changes in topology and hydrophobicity contribute to the differences in substrate accessibility, binding affinities, and stability of enzyme-PLA complexes. The images of the docking complexes of each of the variants (Figure 1) show that part of the polymer chain enters the catalytic pocket adopting different orientations, establishing productive contacts with the −OH group of the catalytic Ser, except for the double mutant N81/94A. In this variant, the PLA chain does not virtually enter the tunnel, and most of the molecule remains on the enzyme’s surface. The zoomed view of the active center of this variant shows that PLA is placed at a distance too large to allow an efficient nucleophilic attack. On the contrary, the other three variants have at least one carbonyl group at a distance <3.5 Å and form a Bürgi–Dunitz angle between 95° and 120°, appropriate for catalysis.^34^

### 3.2 Analysis of Computational Predictions for the Docking Complexes

The hydrolytic activity of the OPE variants toward PLA was used to compare the predictive performances of two *in silico* strategies. TTMD is a computational methodology based on *in silico* thermal titration molecular dynamics simulations, where the stability of the enzyme–substrate complex is evaluated under increasing temperatures.^26,30^ On the other hand, XLPFE is an ML-scoring function developed to predict binding affinity in protein–ligand complexes.^27^ It is primarily used to evaluate and rank poses generated by docking simulations, minimizing the number of poses. The values returned from both approaches are dimensionless and are used to compare the stability and binding affinity predicted for the different OPE-PLA complexes (Table 1). The MS coefficient from TTMD ranges between 0 and 1, with the lowest values indicative of stronger stability, while higher values of the XLPFE score correlate with higher binding affinity.

**Table 1:** Predictions of the Stability (TTMD, MS Coefficient) and Binding Affinity (XLPFE, ML Scoring Function) for the Complexes of the Four OPE Variants with the 3-Mer (PLA_3_) or the 15-Mer (PLA_15_) of d-LA

Method	Substrate	OPE WT	OPE N81A	OPE N94A	OPE N81/94A
TTMD (MS coefficient)	PLA_3_	0.01000	0.05000	0.01667	0.00714
	PLA_15_	0.00490	0.00421	0.00542	0.00575
XLPFE (score)	PLA_3_	4.5408	5.2424	4.6517	4.5172
	PLA_15_	6.9829	7.5412	7.7969	6.8013

The MS coefficients from TTMD simulations were very close to 0, ranging between 0.05 and 0.0042, which points to extremely stable enzyme–substrate interactions in all complexes. However, these values do not guarantee that the substrates are well oriented, as they could be due to nonproductive surface binding, which would also produce very low MS values. According to these predictions, PLA_15_ establishes stronger interactions with the OPE variants than with the short substrate. The predicted stability of PLA_15_–enzyme complexes was greatest for N81A, followed by WT, N94A, and, finally, N81/94A. The coefficients calculated for the complexes with PLA_3_ were between 1.2 and 11 times higher, with enzyme–substrate stabilities ordered very differently. It is interesting to note that complex N81A-PLA_15_ is forecasted as the most stable, being just the opposite for the complex with the short model substrate. One of the factors that affects the stability of a complex is the size of the ligand (the polymeric substrate, in this case); therefore, the reduction in the contact points of PLA_3_ with the enzyme surface may justify this difference. Figures S1 and S2 display the plots for interaction-fingerprint similarity (IFP CS vs time, from which MS coefficients were calculated) and ligand RMSD vs time for both PLA_15_ and PLA_3_ complexes returned from TTMD analysis. RMSD trajectories with PLA_15_ do not differ much among the OPE variants, except for N81/94A, where ligand RMSD rises to 16 Å by the end of the simulation, indicating greater mobility of the substrate. In this last case, the docking model presented in Figure 1 shows that PLA_15_ does not enter the tunnel-shaped catalytic pocket and remains mostly on the protein’s surface, which can justify this predicted higher mobility and the low catalytic efficiency of the double mutant.

When the XLPFE scores computed for the different complexes were compared, the binding affinity was predicted to be higher with PLA_15_ than with PLA_3_. For PLA_15_, the binding affinity ranking was N94A > N81A > WT > N81/94A, and with PLA3, N81A > N94A > WT > N81/94A.

### 3.3 Experimental Validation of Computational Predictions

The four OPE variants were heterologously produced in *K. phaffii* as previously described^18^ in amounts very similar to those documented for the WT enzyme in YEPS medium (Figure S3).^35^ Following FPLC, protein purity was verified via SDS-PAGE (Figure S4). The specific activity against *p*NPB of each of the pure enzymes was consistent with that reported by Payá-Tormo et al.^18^

Benchmarking of the computational predictions was performed by testing the four enzymes on the hydrolysis of real PLA samples. The experimental results (Figure 2) align well with the computational predictions obtained from TTMD simulations. The N81A variant, which had the lowest MS coefficient (0.00421), indicative of the strongest binding affinity, demonstrated the highest hydrolytic activity. This was followed by WT OPE (MS coefficient of 0.0049), which showed weaker binding and correspondingly lower hydrolysis rates. The mutant N94A and the double mutant N81/94A, with the highest MS coefficients (0.00542 and 0.00575), indicative of the weakest binding, were the least effective in PLA hydrolysis. In contrast, when MS coefficients were calculated for a PDLA trimer, there was no correlation with experimental data, which emphasizes that short ligands are insufficient to model the relevant enzyme–polymer interactions, as also reflected in docking (Figure 1). Since PLA is a long, hydrophobic macromolecule, the accuracy of predictive models is highly influenced by ligand length.

**Figure 2:**
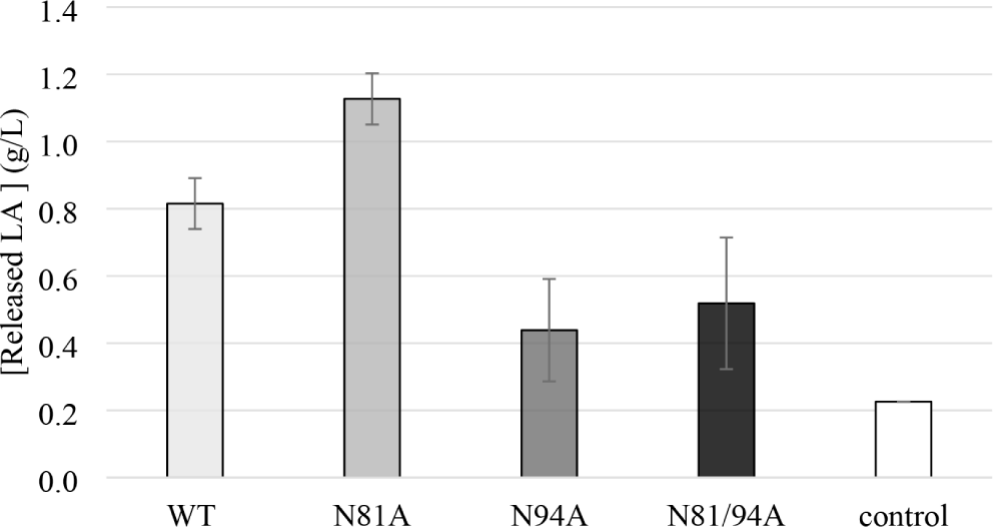
Hydrolysis of 10 g/L 75–120 kDa PLA catalyzed by 0.1 mg/mL purified enzymes. Reactions were kept at 50 °C and 1000 rpm in 100 mM Tris-HCl (pH 7) for 7 days. Reactions without enzymes were set as negative controls.

When it comes to XLPFE scores, the predictions for PLA_15_ also provided valuable insights into the hydrolytic performance of the enzyme variants. The predictions align well with the experimental results for the N81A mutant, with a high XLPFE score (7.5412) and the highest *in vitro* activity, and for the double mutant N81/94A, with low experimental activity and the lowest score (6.8013). This correlation breaks down for N94A, which shows the highest predicted affinity (XLPFE score of 7.7969) but the lowest catalytic activity *in vitro*. Interestingly, WT OPE, with the second-highest hydrolytic activity, had a lower predicted score (6.9829) than N94A and N81A. These results suggest that while XLPFE captures binding affinity well, strong binding alone does not guarantee high catalytic efficiency. XLPFE estimates static affinity and, as well as DiffDock, is trained on small ligands, so their application to polymeric substrates should be made with caution. Also, according to the Sabatier principle, enzyme–substrate (ES) complexes with intermediate stability often result in optimal catalytic activity, as overbinding may hinder substrate turnover or product release, while weak binding may prevent effective catalysis.^36^ The applicability of this principle to the hydrolysis of synthetic polyesters has been recently confirmed, using the depolymerization of PET as an example.^37^ With the short substrate PLA_3_, XLPFE scores were less consistent with experimental trends, and the differences among variants were smaller.

These findings support the predictive value of both TTMD simulations and XLPFE, as the computed enzyme–substrate complex stabilities and binding affinities are consistent with the efficiencies of the experimental hydrolysis. Moreover, docking studies support the hypothesis that increased hydrophobicity, as seen in N81A and N94A, can, in some cases, improve PLA accommodation and subsequent hydrolysis.

## 4 Conclusions

This study demonstrates the effectiveness of combining computational predictions with experimental validation in the field of enzyme engineering. Comparison of the results with short- and long-chain model substrates shows the importance of choosing a long enough substrate to capture well all the potential enzyme–substrate interactions. TTMD was able to predict the binding stability of each OPE variant proportional to experimental PLA depolymerization when PLA_15_ was used as substrate. Similarly, XLPFE scores calculated with PLA_15_ provided insights into the binding affinity of the enzyme–substrate complexes, with mutant N81A and WT OPE having intermediate-to-high predicted binding affinities, consistent with superior experimental performance. Hence, we successfully implemented here for the first time these computational tools to predict the catalytic potential of an enzyme (OPE) and 3 mutated variants, paving the way for the application and refinement of these methodologies in enzyme catalysis.

These *in silico* approaches can be used to reduce the time and resources necessary to identify and characterize enzyme variants with improved catalytic properties. Future rational lid engineering could help tune enzyme–substrate stability to intermediate strength that favors both binding and turnover. Finally, the results presented in this work underscore the critical importance of selecting appropriate model substrates in *in silico* simulations, as excessively simplified analogs can lead to misleading or biologically irrelevant predictions. Future work should include a detailed characterization of the affinity constants of enzymes for the substrate to evaluate the most appropriate algorithms for their prediction.

## Supplementary Material





## Notes

Daily measurements of optical density, protein concentration, and esterase activity in*K. phaffii*cultures (Figure S1); SDS-PAGE of extracellular crude extracts and purified proteins (Figure S2); interaction fingerprints and root mean square deviation of OPE variants with PLA_3_ (Figure S3) and PLA_15_ (Figure S4) (PDF)
Docking complexes (ZIP)

## #

Centro Nacional de Biotecnología (CSIC), 28049 Madrid, Spain
