# Supplementary material for: Computational Study of a Versatile Lipase for the Degradation of Polylactic Acid
Source: ACS Omega. 2025 Nov 20;10(47):57463–8. doi: 10.1021/acsomega.5c07809 (PMC12676354; doi:10.1021/acsomega.5c07809)
Supplement: Supplementary file 1 [file ao5c07809_si_001.pdf]

Computational study of a versatile lipase for the degradation of polylactic acid

Carlos Murguiondo Delgado[a], Mario García de Lacoba[a], Valentina Acosta-Borrero[a#], Jorge Barriuso[a], and Alicia Prieto[a]**

[a]Centro de Investigaciones Biológicas Margarita Salas (CIB-CSIC), Ramiro de Maeztu 9, Madrid, Community of Madrid, ES 28040

*Email: aliprieto@cib.csic.es

*Email: jbarriuso@cib.csic.es

KEYWORDS. Polylactide (PLA), bioplastics, Thermal Titration Molecular Dynamics, XLPFE, docking, lipase.

a) OPE WT

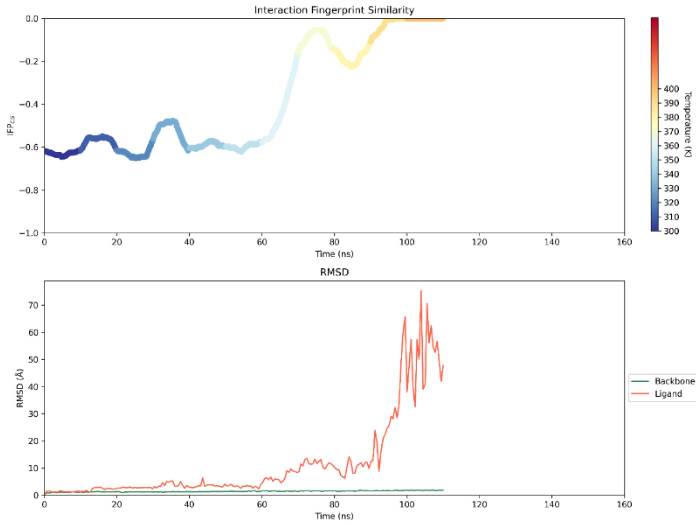

b) OPE N81A

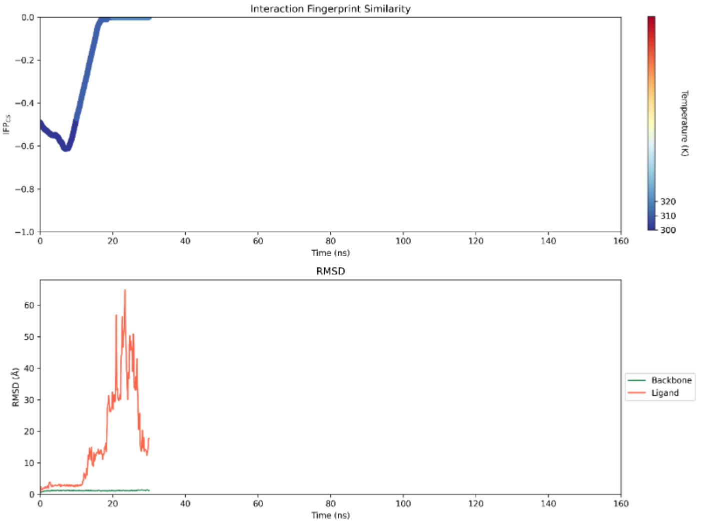

c) OPE N94A

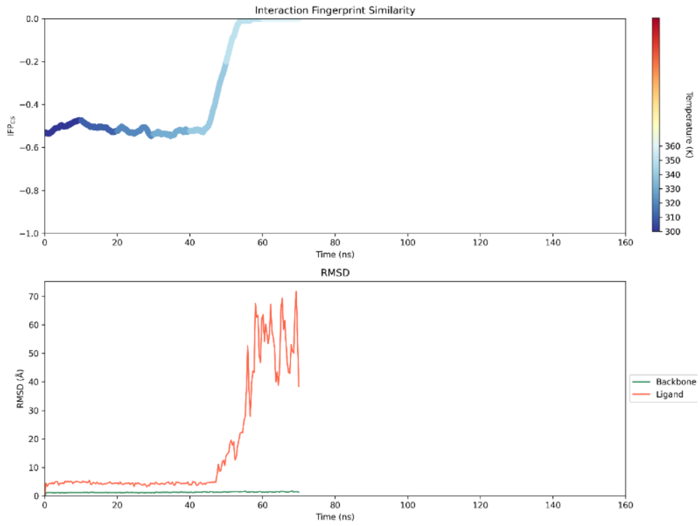

d) OPE N81/94A

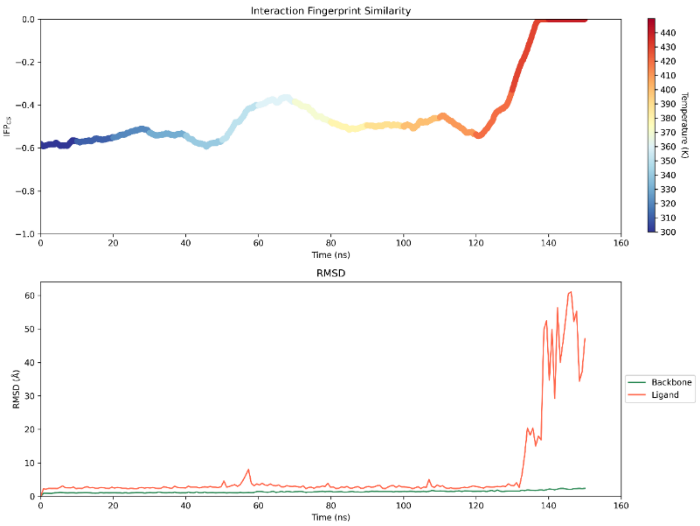

Figure S1. IFP-CS and RMSD time traces for OPE variants with PLA_3. Interaction-fingerprint similarity (IFP-CS, top panels) and root-mean-square deviation (RMSD, bottom panels) are shown for the four enzyme–ligand complexes: (a) OPE WT, (b) OPE N81A, (c) OPE N94A, and (d) OPE N81/94A. Traces are taken from representative TTMD production trajectories (160 ns; integration timestep 2 fs).

a) OPE WT

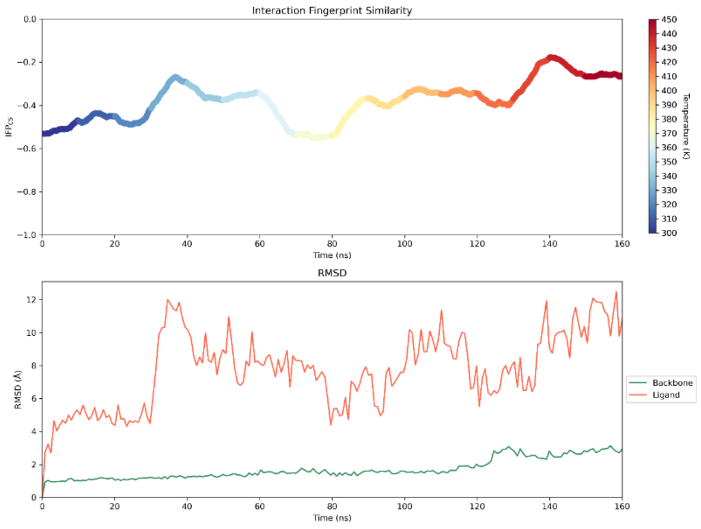

b) OPE N81A

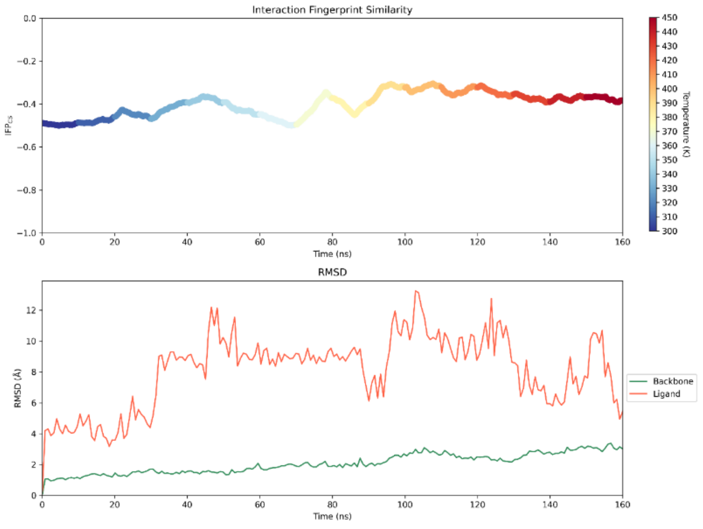

c) OPE N94A

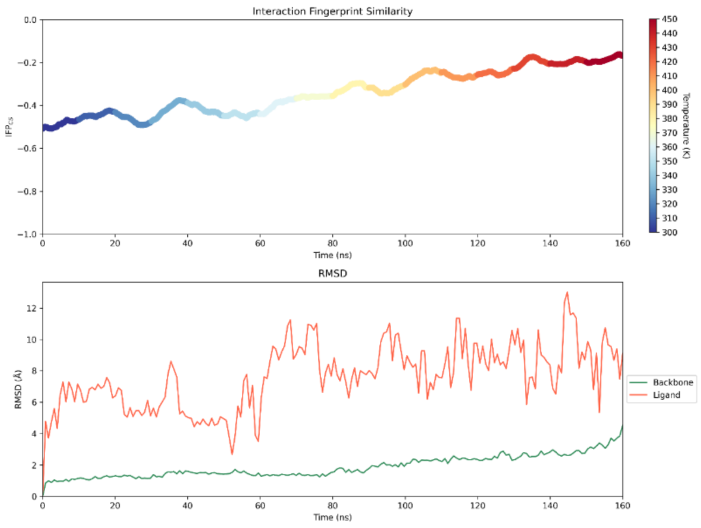

d) OPE N81/94A

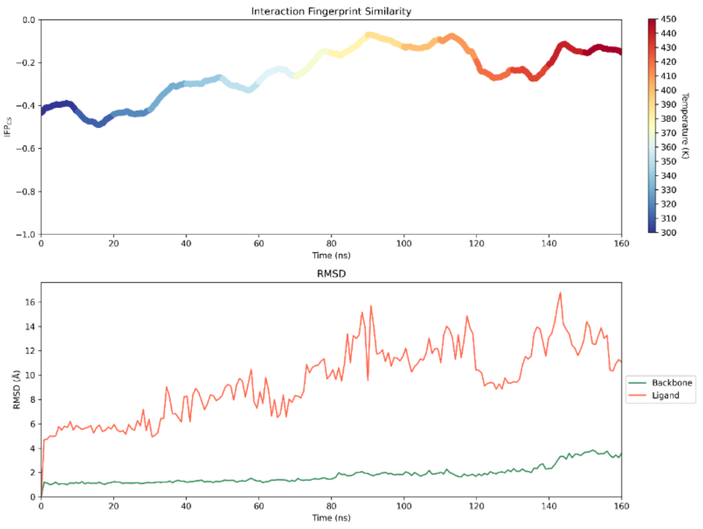

Figure S2. IFP-CS and RMSD time traces for OPE variants with PLA_{15}. Interaction-fingerprint similarity (IFP-CS, top panels) and root-mean-square deviation (RMSD, bottom panels) are shown for the four enzyme–ligand complexes: (a) OPE WT, (b) OPE N81A, (c) OPE N94A, and (d) OPE N81/94A. Traces are taken from representative TTMD production trajectories (160 ns; integration timestep 2 fs).

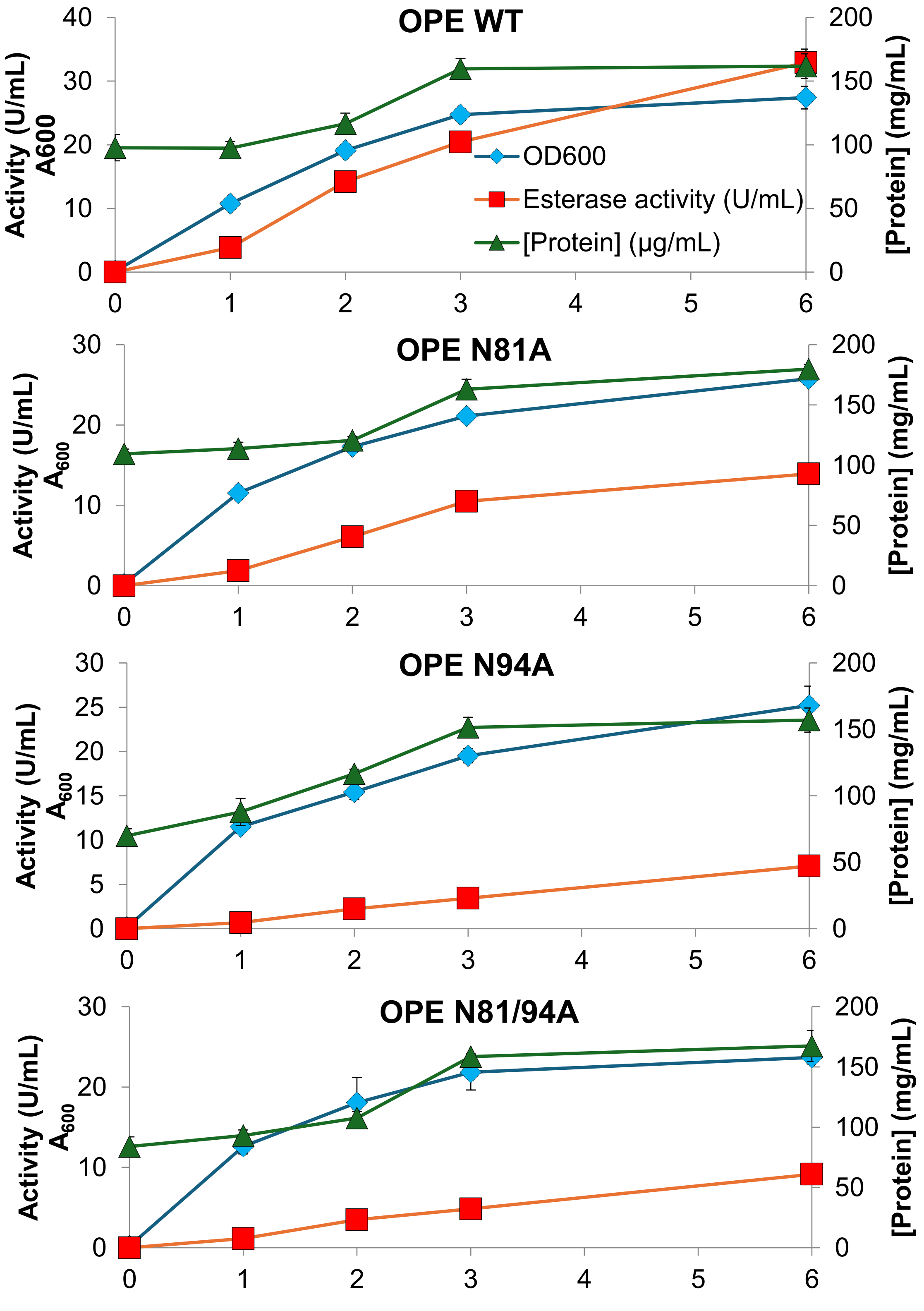

Figure S3. Optical density (A_{600}), extracellular protein concentration and esterase activity measured in *K. phaffii* cultures over 6 days. Cultures were grown in YEPS medium at 28 ºC and 250 rpm, with the addition of 0.5% methanol (w/v) every 24 h.

70 kDa

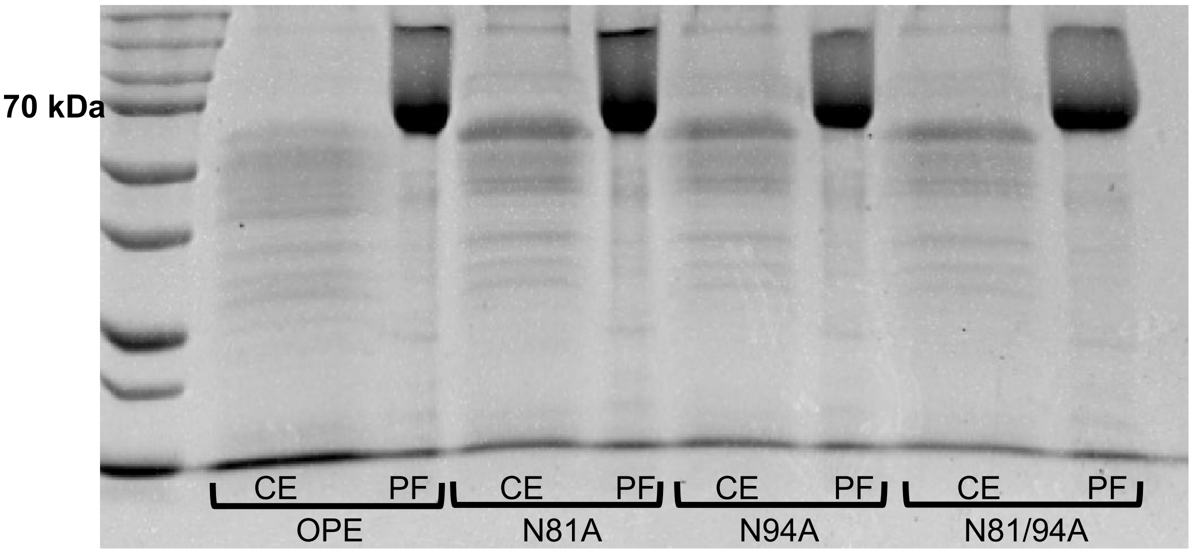

Figure S4. SDS-PAGE analysis of extracellular crude extracts (CE) and purified fractions (PF) of the enzymes.
